# Ultrasound-guided platelet-rich plasma injections for post-traumatic greater occipital neuralgia: study protocol for a pilot randomized controlled trial

**DOI:** 10.1186/s40814-021-00867-3

**Published:** 2021-06-22

**Authors:** Jacqueline E. Stone, Tak S. Fung, Matthew Machan, Christina Campbell, Rodney Li Pi Shan, Chantel T. Debert

**Affiliations:** 1 Department of Clinical Neurosciences, Division of Physical Medicine and Rehabilitation, University of Calgary, 1403 29 Street NW, Calgary, Alberta T2N 2T9 Canada; 2 Information Technologies, University of Calgary, Calgary, Alberta Canada

**Keywords:** Greater occipital neuralgia, Post-traumatic headaches, Concussion, Traumatic brain injury, Platelet-rich plasma, Corticosteroids, Ultrasound guidance, Randomized controlled trial

## Abstract

**Background:**

Post-traumatic headaches (PTH) are a common sequelae of traumatic brain injury (TBI) and greatly impact patient function and quality of life. Post-traumatic greater occipital neuralgia (GON) is a type of post-traumatic headache. Conventional treatment includes steroid/anesthetic injections which typically alleviate pain but have a short duration of effect. Platelet-rich plasma (PRP) is an emerging biological treatment for numerous degenerative disorders, including peripheral nerve disorders. The primary aim of this pilot study is to evaluate whether a randomized control trial of PRP for the treatment of GON in patients with post-traumatic headaches is feasible in regard to recruitment, adherence, retention, and adherence and adverse events. Exploratory aims include improvement in pain, function, and quality of life in patients with post-traumatic GON receiving PRP compared to steroid/anesthetic and normal saline injections.

**Methods:**

Thirty adults (over 18 years of age) with post-traumatic GON will be randomized into one of three groups: (1) autologous PRP injection, (2) steroid/anesthetic injection (standard care), or (3) placebo injection with normal saline. Injections will be performed to the greater occipital nerve under ultrasound guidance by a trained physician. Daily headache intensity and frequency data will be collected pre-injection and for the duration of the study period. Feasibility will be defined as greater than 30% recruitment, 70% completion of intervention, 70% retention, and less than 2 minor adverse events. Exploratory outcomes will be explored using the Headache Impact Test-6 (HIT-6, a valid and reliable 6-item questionnaire for assessment of the impact of headaches across different diagnostic groups of headaches) and the quality of life in following brain injury questionnaire (QOILIBRI).

**Discussion:**

This pilot study will be the first to evaluate the feasibility of PRP as a potential treatment of GON in patients with post-traumatic headache.

**Trial registration:**

ClinicalTrials.gov - NCT04051203 (registered August 9, 2019).

## Background

Traumatic brain injury (TBI) is a leading cause of death and disability [1]. Each year, an estimated 69 million people suffer from TBI worldwide, accounting for significant economic burden, with costs exceeding $76.5 billion ($85.1 in 2020 dollars) in the USA alone [2]. There are many medical sequelae following TBI that greatly impact an individual’s function and quality of life. Headache is one of the most common complaints, affecting up to 71% of TBI patients in the first year after injury, with headache symptoms persisting up to 10 years in some cases [3–5]. To date, the causal mechanisms of post-traumatic headache (PTH) have not been fully characterized, thereby limiting therapeutic targets.

PTH is a secondary headache disorder that can present with features of any primary headache disorder, and diagnostic criteria are defined by International Classification of Headache Disorders 3rd Edition (ICHD-3) [6]. Occipital neuralgia (ON) is a primary headache disorder, that can be seen commonly in the post-traumatic setting. ON is defined as unilateral or bilateral paroxysmal, shooting or stabbing pain in the distribution(s) of the greater, lesser, and/or third occipital nerves [6]. ON is most commonly unilateral (85%) and involves the greater occipital nerve in 90% of cases [7]. The etiology of greater occipital neuralgia (GON) is not fully understood, but damage and irritation along the course of the greater occipital nerve are believed to play a primary role in its pathogenesis [8]. Increased incidence following posterior head trauma, whiplash injury, and helmet use has been reported [9–11]. Patients with GON are typically treated with local greater occipital nerve blockade (cortisone and anesthetic), as it has the potential of being both diagnostic and therapeutic [6, 12].

Although greater occipital nerve blockade is effective in relieving headaches and associated pain, these effects are transient, with a mean duration of pain relief of just 1 month [13]. Furthermore, the risks and complications of repeated steroid injection such as weight gain, glycemic abnormalities, tissue necrosis, and hypothalamic-pituitary-adrenal axis suppression, make it less favorable as a long-term treatment [14, 15]. Other treatments described in the literature include systemic medications (i.e., NSAIDS, anti-depressants, anti-epileptics), botulinum toxin, pulsed radiofrequency ablation, occipital nerve stimulation, and surgical decompression [16, 17]. Unfortunately, these treatments have transient and widely variable success rates, highlighting the need for new and effective therapies.

Platelet-rich plasma (PRP) is an emerging biological treatment modality containing supraphysiologic concentrations of platelets, plasma, and associated growth factors [18]. PRP has garnered widespread interest as a safe and effective treatment modality in multiple fields, including orthopedics, sports medicine, ophthalmology, neurosurgery, and plastic surgery [19]. Given its efficacy in treating numerous degenerative and inflammatory conditions, PRP has recently been highlighted as a potential treatment for peripheral neuropathies [20, 21].

PRP’s anti-inflammatory and regenerative properties, safety profile, and longer duration of effect make it an attractive therapeutic modality over conventional steroid treatment in peripheral nerve disorders. PRP augments the biological repair process through several mechanisms. Specifically, it acts to encourage local angiogenesis, augment inflammation, inhibit catabolic cytokines, recruit local stem cells and fibroblasts, and induce local manufacturing of growth factors involved in tissue repair [21, 22].

Multiple animal and in vitro studies have demonstrated PRP’s ability to assist in remyelination, axonal regeneration, and functional neurologic recovery in models of peripheral nerve injury [20, 23–32]. There have been few in vivo studies of PRP in treatment of peripheral neuropathies with variable success rates [33–37]. One randomized controlled trial found significant pain reductions in 60 patients with carpal tunnel syndrome and improvement of multiple markers of nerve function which persisted to 6 months following a single PRP injection [20]. Another clinical trial found significant improvement in pain following perineural PRP injections in patients with diabetic polyneuropathy [30].

These findings are encouraging as they reveal new therapeutic avenues in the management of peripheral nerve disorders. To our knowledge, PRP has not been investigated as a treatment for GON. We believe that PRP’s physiology, demonstrated efficacy, and safety profile make it an exciting and novel strategy to treat this debilitating condition. This study will investigate the feasibility of a randomized control pilot study for the treatment of GON in patients with post-traumatic headache using PRP. This pilot trial will evaluate the ability to recruit patients, retain patients throughout the protocol and the feasibility of implementing randomization using PRP for the treatment of patients suffering with GON following TBI. Our specific objectives are the following:

1. Evaluate the feasibility of a randomized pilot study of PRP as a treatment for patients with GON and post-traumatic headache in terms of recruitment (greater than 30%), attendance (70% intervention appointment attendance), retention (greater than 70% complete protocol), and acceptability of the protocol.
2. Evaluate the safety profile of PRP for the treatment of GON in patients with post-traumatic headaches with less than 2 minimal adverse events.
3. Exploratory objectives include evaluating whether patients with GON and post-traumatic headaches receiving PRP have significantly decreases pain, improved function, and quality of life, compared to patients receiving cortisone/anesthetic and normal saline injections.

## Methods

### Study design

**Fig. 1:**
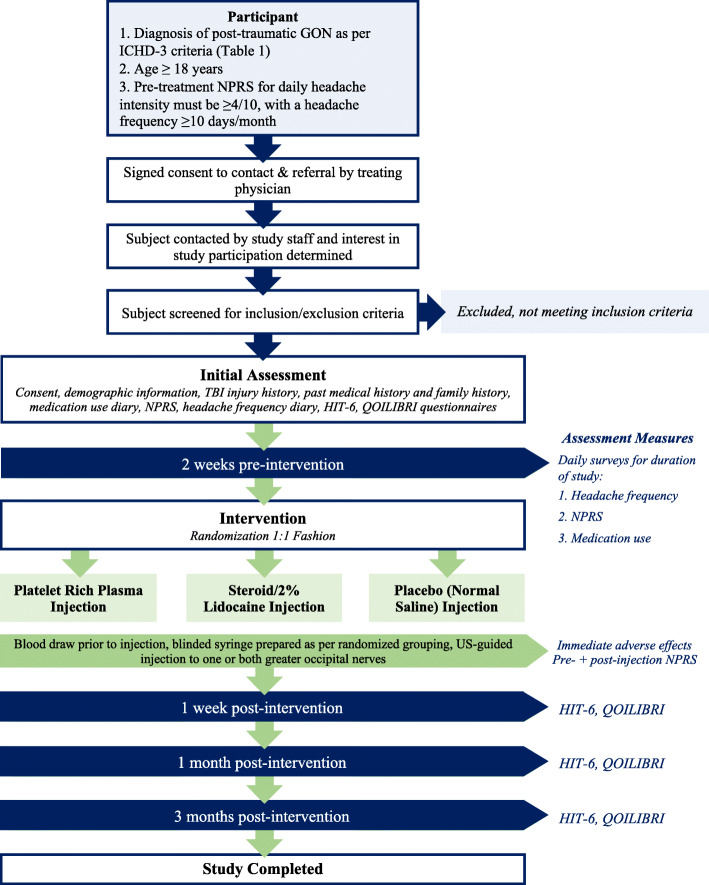
Study design

Prospective, randomized, controlled, double-blinded pilot trial evaluating the feasibility in relation to recruitment, retention, and acceptability of a single perineural PRP injection compared to that of steroid/anesthetic injection or injection with normal saline to the greater occipital nerve as a treatment for GON in patients following TBI. Flow through the study is portrayed in Fig. 1.

### Study registration

The trial protocol is registered on ClinicalTrials.gov (NCT04051203). This study was registered at ClinicalTrials.gov on August 9, 2019; the study was open for enrollment at this time. This study protocol was prepared in accordance with the SPIRIT (Standard Protocol Items: Recommendations for Interventional Trials) guidelines for reporting clinical trials [38].

### Study setting and recruitment

Participants will be recruited primarily from the Calgary Brain Injury Program at the Foothills Medical Centre. Recruitment in this study will also be advertised at other Calgary-area neurology and sports medicine clinics. Once a participant is referred and has provided consent to contact, they will be contacted by a member of our research team. If they meet inclusion criteria at that time, an initial assessment will be scheduled and participants will complete a digital informed consent.

### Study participants

#### Inclusion criteria

Eligible participants will be males or females at least 18 years of age who suffer from post-traumatic headaches secondary to GON. Patients must fulfill the ICHD-3 criteria [6] for post-traumatic headache (Table 1) and for GON (Table 2) in establishing a diagnosis of post-traumatic GON. This diagnosis will be established by an experienced physiatrist and/or neurologist with extensive experience in headache and related disorders. To meet this criteria, participants must have experienced previous successful temporary relief with local anesthetic/steroid injection surrounding the greater occipital nerve in the past, but have not received local injection within the past 3 months. Pre-treatment numerical pain rating scale (NPRS) for daily headache intensity must be ≥ 4/10, with a headache frequency ≥ 10 days/month. Possible secondary causes of headache must be ruled out with a reasonable level of investigation prior to enrollment.

**Table 1:** The International Classification of Headache Disorders 3^rd^ Edition (ICHD-3) diagnostic criteria for persistent headache attributed to traumatic injury to the head [6]

A.	Any headache fulfilling criteria C and D
B.	Traumatic injury to the head has occurred
C.	Headache is reported to have developed within 7 days after one of the following: 1. The injury to the head 2. Regaining consciousness following injury to the head 3. Discontinuation of medication(s) impairing ability to sense or report headache following injury to the head
D.	Headache persists for > 3 months after its onset
E.	Not better accounted for by another ICHD-3 diagnosis

**Table 2:** The International Classification of Headache Disorders 3^rd^ Edition (ICHD-3) diagnostic criteria for occipital neuralgia [6]

A.	Unilateral or bilateral pain in the distribution(s) of the greater, lesser, and/or third occipital nerves and fulfilling criteria B-D
B.	Pain has at least two of the following three characteristics: 1. Recurring in paroxysmal attacks lasting from a few seconds to minutes 2. Severe in intensity 3. Shooting, stabbing, or sharp in quality
C.	Pain is associated with both of the following: 4. Dysesthesia and/or allodynia apparent during innocuous stimulation of the scalp and/or hair 5. Either or both of the following: a. Tenderness over the affected nerve branches b. Trigger points at the emergence of the greater occipital nerve or in the distribution of C2
D.	Pain is eased temporarily by local anesthetic block of the affected nerve(s)
E.	Not better accounted for by another ICHD-3 diagnosis

#### Exclusion criteria

Inability to provide informed consent; history of surgery in the occipital region; unstable psychiatric or medical condition; uncontrolled rheumatologic or inflammatory disorders; widespread neurologic disorders (e.g., MS); fibromyalgia/chronic fatigue syndrome; coagulopathy; immunosuppression; active cancer; herpes zoster infection in last 6 months; pregnancy; steroid injection to the greater or lesser occipital nerve infiltration in past 3 months.

### Blinding and randomization

Each participant will be randomized following screening and enrollment in the study. Participants will be randomized (via sealed envelope) by a blinded research assistant in a 1:1 fashion to one of three treatment arms: (1) autologous PRP injection, (2) steroid/anesthetic injection (standard care), or (3) placebo injection with normal saline. All patients will undergo 60 mL blood draw on their scheduled day of injection. Whole blood samples will be prepared in the PRP group only; otherwise, they will be discarded appropriately. Syringes (3 mL each) will be prepared by a research assistant and will be covered in an opaque tape so the physician providing the injection is blinded to the type of injectate. The physician will fill out a questionnaire at the time of injection indicating their best guess of the syringe contents, which will be evaluated afterwards, to ensure adequate blinding. Unblinding will occur only in extraordinary circumstances if knowledge of the actual treatment received is deemed essential to providing further patient care.

### Interventions

Injections will be prepared as stated below. Patients will be asked to complete a numeric pain rating scale (NPRS) immediately pre- and post-injection. Patients will be asked to refrain from use of anti-inflammatory medications for 2 weeks prior to the injection and for 2 weeks following the injection due to the possible inhibitory effect on the action of PRP. Two 3 mL syringes with 2 mL of injectate (PRP, steroid/anesthetic, or normal saline) will be prepared for each participant. Patients will be assessed at the time of injection and will receive a single injection, if experiencing unilateral symptoms only, or two injections to each greater occipital nerve, if symptoms are bilateral. Patients will be monitored for 30 min following injection for any immediate adverse reactions, such as local reaction, increased pain intensity, anaphylaxis, nausea, or dizziness.

#### Platelet-rich plasma

PRP will be prepared using the “Arthrex Angel System,” which is a fully automated PRP preparation machine. Sixty milliliters of blood will be drawn from the antecubital vein and processed via centrifugation. Five milliliters of sodium citrate will be added to the syringe prior to blood draw to prevent coagulation. Samples will be centrifuged as per manufacturer instructions, yielding 5 mL of PRP. For quality testing, 1 mL of PRP will be sent to the lab for analysis of platelet and leukocyte count, as compared to the patients’ whole blood. The remaining 4 mL of PRP will be divided into two syringes and 2 mL will be injected per symptomatic side.

#### Steroid/anesthetic

Steroid injections will be prepared to include 20 mg Depo-Medrol and 2 mL 2% lidocaine.

#### Normal saline

Placebo injections will be with 2 mL normal saline.

### Injection technique

Given that ultrasound (US)-guided greater occipital nerve blockade demonstrated superiority over conventional blind technique in one study [39], participants will receive perineural injections under ultrasound guidance. Injections will be performed bilaterally if participants’ symptoms are bilateral; otherwise, they will be performed unilaterally. Where the greater occipital nerve is not visible under US, injection will be performed in accordance with conventional blind technique [40].

### Measures

Demographic information will be collected 2 weeks prior to starting the study including age, sex, weight/height, education, family history, past medical history, and medication use. Headache history will be collected including headache frequency, severity, as needed medication-use, type of headache, associated symptoms (i.e., paresthesia, fronto-orbital pain, nausea, vomiting), and headache triggers.

Questionnaires will be completed 2 weeks prior to injections, 1 week, 1 month, and 3 months post-injection. A daily headache diary provided via mobile application (Secure RedCap) available in iPhone or android device will be provided to record daily records of NPRS, headache frequency, and medication-use. Patients will complete daily headache diaries beginning at 2 weeks pre-injection and ending 3 months post-injection. See Figure 1 for a schematic of the study design.

### Primary outcomes

The primary outcomes of this trial will assess the feasibility of a double-blinded randomized control trial for the treatment of GON in patients with post-traumatic headaches. This pilot trial is a smaller version and will aim to inform a more extensive and larger version of this randomized control trial. Specific outcomes for evaluation will include the following:

1. Recruitment: Ability to recruit at least 30% of those patients screened for participation.
2. Intervention attendance: At least 70% of those patients recruited for the study will attend and participate in the primary intervention.
3. Retention: At least 70% of those receiving the primary intervention will complete the 3-month follow-up.
4. Adverse events: Adverse events will be minimal. No more than 1 individual will have an adverse event. Any adverse event will be minor and reversible (i.e., nausea, light headedness, temporary pain at site of injection).

### Exploratory outcomes

The primary exploratory outcome will be a reduction in the numeric pain rating scale (NPRS) at 3 months in the PRP group, as compared to the steroid and placebo groups. The suggested minimal clinical important difference (MCID) at the time of writing is 2 points [41] or ≥ 50% compared to placebo. Secondary outcomes will include headache frequency based on daily diaries and NPRS. Additional questionnaires completed at 2 weeks prior to injection, 1 week, 1 month, and 3 months post-injection will be the Headache Impact Test-6 (HIT-6; a valid and reliable 6-item questionnaire for assessment of the impact of headaches across different diagnostic groups of headaches [42, 43]) and the quality of life in following brain injury questionnaire (QOILIBRI). As well, participants will complete daily medication diaries tracking prn analgesic use.

### Attrition and adherence

Participants will be withdrawn from the study if there is a change in routine medications during the study period or if they do not complete study questionnaires. Daily headache diaries will be completed by all participants for the duration of the study submitted on their mobile device. In the event that daily diaries are not completed, reminders will be sent out by the study team.

### Data management and monitoring

Participants will be assigned a study ID at the time of study enrolment. All identifying information will be removed once study participant numbers have been assigned and data is collected. Study data will be entered into a highly secure electronic REDCap database (REDCap 7.6.9 2020 @ Vanderbilt University). Only research team members will have access to this database. Paper copies of any data or participant information will be stored in a secure fashion. All data will be retained for 5 years following project completion in accordance with the University of Calgary Conjoint Health Research Ethics Board.

There will not be a formal data monitoring committee; a research assistant will periodically evaluate participant data for completeness and inform investigators of any issues. Adverse events will be reported via telephone or email, a research assistant will contact participants for further details at the time of reporting. A study physician will be alerted of any serious adverse events that require immediate attention.

### Data analysis

The primary outcomes for this pilot trial require descriptive statistics for recruitment, completion of primary assessment, retention, and adverse effect profiles. For exploratory outcomes, change in NPRS, HIT-6, and QOLIBRI at 1 and 3 months following intervention for patients receiving PRP compared to steroid/anesthetic and normal saline will be analyzed. A two-way mixed ANOVA test will be completed to evaluate the change in NRPS between groups. Chi-square testing will be performed to determine the relationship between basic characteristics in the three groups. For secondary outcomes, Kruskal-Wallis non-parametric analysis based on ranks will be completed correcting for multiple comparisons with Bonferroni correction. All results will be presented with 95% confidence intervals. Results will be preliminary and should be interpreted with caution due to the small sample size.

### Study status

At the time of submission, we are recruiting and enrolling participants in the study.

### Protocol amendments

Any modifications to the study protocol will be submitted and approved through the University of Calgary Conjoint Health Research Ethics Board. The ClinicalTrials.gov registry will be updated as required, and trial participants will be notified of relevant study modifications.

### Access to data

The principal investigator, research assistants, students, and statistician colleagues who are directly involved in the study will have access to the final data set.

### Dissemination policy

Trial results will be disseminated through presentations at conferences, invited presentations, and published manuscripts by study authors and contributors. The study is registered on clinicaltrials.gov. There will be no use of professional writers.

## Discussion

GON in the post-traumatic setting is a debilitating condition without effective long-term treatment options. GON can be seen commonly following concussion, especially in cases of posterior head trauma or associated whiplash injury. PRP has been studied extensively across multiple degenerative conditions but has only recently been evaluated as a potential treatment for peripheral neuralgias. PRP has the proposed advantage of restoring normal nerve physiology and spares the use of steroids, which are associated with multiple adverse effects, especially when provided repeatedly over the long term. In addition to improved management of chronic pain, patients may experience enhanced function, quality of life, and reduced medication usage. This pilot study will provide evidence to explore whether a larger double-blinded randomized controlled trial for the treatment of GON in patients with post-traumatic headache with PRP is feasible. Feasibility will be based on successful recruitment, completion of the primary intervention, retention, and adverse events.

## Data Availability

Not applicable.

## Abbreviations

GON: Greater occipital neuralgia
HIT-6: Headache impact test
ICHD: International classification of headache disorders
MCID: Minimal clinical important difference
NPRS: Numerical pain rating scale
PRP: Platelet-rich plasma
PTH: Post-traumatic headache
QOILIBRI: Quality of life in following brain injury questionnaire
TBI: Traumatic brain injury
US: Ultrasound
